# Study of the Therapeutic Effects of Chinese Herbal Decoction Combined with Glucocorticoid in Treating Primary Nephrotic Syndrome in Children

**DOI:** 10.1155/2021/4434504

**Published:** 2021-11-19

**Authors:** Xue Hou, Meihua Xu, Jie Li, Rui Li, Jinzhi Zhang, Jing Ju

**Affiliations:** ^1^Department of Clinical Laboratory, Yantaishan Hospital, Yantai 264000, China; ^2^Department of Pharmacy, Traditional Chinese Medical Hospital of Huangdao District, Qingdao 266500, China; ^3^Department of Emergency, Qingdao Hospital of Traditional Chinese Medicine, Qingdao Hiser Hospital, Qingdao 266033, China; ^4^Department of Endocrinology, Zhangqiu District People's Hospital, Jinan 250200, China; ^5^Department of Imaging, Zhangqiu District People's Hospital, Jinan 250200, China; ^6^Outpatient Department, Weifang People's Hospital, Weifang 261041, China

## Abstract

**Background:**

To investigate the clinical effects of Chinese medicine decoction combined with glucocorticoid in treating children with primary nephrotic syndrome.

**Methods:**

A total of 70 children with pediatric nephritis nephrotic syndrome treated at Weifang People's Hospital from January 2019 to December 2019 were randomly allocated to the therapy group and the control group, each with 35 cases. The control group was treated with conventional Western medicine, and the therapy group received Western medicine and Chinese medicine. After 12 weeks of treatment, the therapeutic effect of the two groups was compared.

**Results:**

After receiving the treatment, the levels of urine protein (UPro), triglyceride, and cholesterol were significantly decreased in the two groups (*p* < 0.05), and these levels in the therapy group were much lower than those of the control group (*p* < 0.05). However, the level of albumin (ALB) was predominantly increased in the two groups after treatment (*p* < 0.05), and this level in the therapy group was much higher than that of the control group (*p* < 0.05). Moreover, the immune indicators, coagulation function, and recurrence rate were noticeably improved after treatment (*p* < 0.05), and the therapy group was better than the control group (*p* < 0.05). Furthermore, the comparison of renal function indexes, liver function indexes, and blood routine between the two groups showed no statistical significance in the incidence of adverse reactions between the two groups (*p* > 0.05).

**Conclusions:**

For the treatment of refractory nephrotic syndrome in children, based on conventional shock therapy, the addition of traditional Chinese medicine (Liuwei Dihuang pill decoction) remedy can significantly improve the disease symptoms in children and improve the efficacy, and the incidence of adverse reactions is low.

## 1. Introduction

Primary nephrotic syndrome (PNS) is a syndrome of glomerular diseases caused by a variety of etiologies, with increased levels of proteinuria (>3.5 g/24 h), hypoalbuminemia (serum albumin<30 g/L), and hyperlipidemia and varying degrees of edema being the main manifestations of the clinical syndrome [1, 2]. The course of childhood nephrotic syndrome is protracted and difficult to heal, and it can enter adulthood at the longest. Due to incomplete physiological development and poor immunity, children with this condition are highly susceptible to infection during the treatment process, which can lead to severe kidney disease [3–5], which seriously threatens the patient's life and safety and causes economic and psychological problems to the patient. As a result, the quality of life is greatly affected. Therefore, effective treatment of this condition to delay its progress has become an urgent need.

Current medical treatment of pediatric nephrotic syndrome includes glucocorticoids [6, 7], immunosuppressants, and immunomodulators, among which glucocorticoids are the drugs of choice for nephrotic syndrome. Although it can effectively reduce protein exudation by controlling the occurrence of the body's inflammatory reaction, its immunosuppressive effects could cause infection, osteoporosis, gastrointestinal reactions, lipid metabolism disorders and liver enzyme abnormalities, and other adverse reactions [8]. Most pediatric patients with primary nephrotic syndrome are relieved after initial hormone therapy, and about 85% of the children relapse after the initial treatment remission, of which 25%–43% have frequent recurrences [9, 10]. Several clinical studies have reported [11] that 80% to 90% of children with primary nephrotic syndrome are sensitive to glucocorticoids and can be relieved by hormone therapy, while the rest are hormone-resistant. In addition, 50% of hormone-sensitive children will have hormone dependence or recurrent illnesses. If the symptoms are not relieved timely, serious complications may occur or even can lead to end-stage renal disease [10].

Because this disease has the characteristics of recurrence and relapse easily, coupled with poor patient compliance, it becomes challenging to achieve the desired outcomes. The use of traditional Chinese medicine (TCM) can not only reduce the side effects of hormones, increase the sensitivity of hormones, and inhibit the recurrence of the disease, but also improve the overall physical condition of the patient and improve the prognosis of the patient [9, 12, 13]. TCM occupies an indispensable and important position in the treatment of this disease. It is clinically proven that TCM treatment of nephrotic syndrome can turn urine protein into negative, reduce the use of hormones and side effects, and improve the quality of life of children with nephropathy. Therefore, through clinical investigation in children with recurrent nephrotic syndrome based on TCM syndrome differentiation, analysis of their prescription medication rules, analysis of appropriate prescriptions, and selection of therapeutic drugs, the experience is summarized for TCM syndrome differentiation and treatment of nephrotic syndrome so as to better guide the clinical application of TCM approaches.

Recently, there have been few reports on the clinical application and effect evaluation of TCM for primary nephrotic syndrome. This article was aimed to explore further the clinical effects of TCM decoction combined with glucocorticoids in the treatment of primary nephrotic syndrome, understand in detail the specific situation of Chinese medicine decoction in the treatment of primary nephrotic syndrome in reducing the side effects of hormones, evaluate its clinical application effect, discuss the advantages of its treatment methods, and provide a potential reference for further improving and promoting its clinical application.

## 2. Materials and Methods

### 2.1. General Information and Medical Records

#### 2.1.1. Research Objects

A total of 70 children with pediatric nephritis nephrotic syndrome treated at the pediatric outpatient clinic or inpatient department of the Weifang People's Hospital, Weifang, Shandong, China, from January to December 2019 were selected. Among them, 48 were inpatient medical records and 22 were outpatient medical records, all diagnosed with nephretotic syndrome. Among them, 52 were male children and 18 were female children, with a male-to-female ratio of 2.9 : 1. The age distribution was 41 cases from 2 to 5 years old, 17 cases from 6 to 9 years old, 9 cases from 10 to 13 years old, and 3 cases from 14 to 18 years old; the youngest age was 2 years old, the oldest age was 17 years old, and the average age was 6.14 years old. At the time of treatment, the shortest course of illness was 3 days and the longest was 8 years.

#### 2.1.2. Diagnostic Criteria

*Western Medicine Diagnostic Criteria*. According to the diagnostic criteria of pediatric primary nephrotic syndrome formulated by the Nephrology Group of the Pediatric Branch of the Chinese Medical Association in 2000 at the Zhuhai Conference [14] and the “Hormone Sensitivity/Relapse” formulated by the Nephrology Group of the Pediatric Branch of the Chinese Medical Association in 2016 relying on the diagnostic criteria of the Evidence-Based Guidelines for Diagnosis and Treatment of Nephrotic Syndrome, the diagnostic criteria are as follows: (1) massive proteinuria: 3 qualitative urine protein in one week (+++)～(++++) or random or morning urine protein/creatinine (mg/mg) ≥2.0; 24 h urine protein quantitative ≥50 mg/kg. (2) Hypoalbuminemia: plasma albumin is less than 25 g/L. (3) Hyperlipidemia: plasma cholesterol is higher than 5.7 mmol/L. (4) Different degrees of edema. (5) Exclude secondary nephrotic syndrome and congenital nephrotic syndromes, such as diabetic nephropathy and lupus nephritis. (6) Frequent recurrence of primary nephrotic syndrome: primary nephrotic syndrome during the disease, there are more than 2 recurrences within half a year, and more than 4 recurrences within 1 year (nephrotic syndrome recurrence: consecutive 3 days, morning urine protein changes from negative to 3+ or 4+, or morning urine uPCR (urine protein/creatinine) ≥2 g/g, or 24 h urine protein quantitative ≥50 mg/kg). The diagnosis can be made on those who have the above (1), (2), (5), and (6) criteria. *Diagnostic Criteria of Chinese Medicine*. This standard refers to the “Guiding Principles for Clinical Research of New Chinese Medicines (Trial)” issued by the National Medical Products Administration in 2002 and “Diagnosis, Syndrome Differentiation and Efficacy Evaluation of Primary Nephrotic Syndrome” issued by the Nephropathy Branch of the Chinese Society of Chinese Medicine in 2006.

#### 2.1.3. Inclusion Criteria

Inclusion criteria were defined as follows: (1) meet the diagnostic criteria of Western medicine; (2) meet the diagnostic criteria of Chinese medicine; (3) children aged 1–18 years old; (4) oral hormones or additional immunosuppressive therapy, and the prescribed treatment course of hormones is stopped after half a year; (5) accepting treatment voluntarily and having a guardian sign the “Informed Consent”; (6) no children with serious diseases such as cardiovascular, liver, and hematopoietic system; and (7) regular follow-up visits and telephone follow-ups.

#### 2.1.4. Exclusion Criteria

Exclusion criteria were defined as follows: (1) congenital and secondary nephrotic syndrome; (2) patients with severe diseases such as cardiovascular, liver, hematopoietic system, etc; (3) those who have participated in clinical research of other drugs within the last 1 month; (4) those who are allergic to Chinese medicine; (5) children who are <2 years old and >18 years old; (6) patients with mental illness; and (7) those who fail to use the prescribed drugs and cannot determine the efficacy.

#### 2.1.5. Case Dropout or Termination Criteria

Case dropout or termination criteria were defined as follows: (1) those who did not meet the inclusion criteria and were mistakenly included; (2) those who meet the inclusion criteria but fail to take the prescribed medication after inclusion; (3) those with severe adverse reactions, serious adverse events, complications or special physiological changes, who are not suitable to continue the trial, or quit by themselves during the trial; (4) cases in which the patients who have not ended the treatment for various reasons automatically withdraw from the trial, are lost to follow-up or die, and the data is incomplete, which affects the investigation; and (5) if the condition deteriorates during the course of the disease, and the clinical trial should be terminated according to the clinician's judgment, the clinical trial of the case should be terminated immediately. During the trial process, it was discovered that the trial protocol had major mistakes and the effectiveness of the treatment plan could not be evaluated for various reasons.

#### 2.1.6. Grouping

Seventy patients who met the criteria for inclusion and exclusion were randomly divided into two groups, namely, the control group: hormone therapy alone, and the therapy group: TCM combined with hormone therapy, with 35 cases in each group. This study was approved by the ethics committee of the Weifang People's Hospital, Weifang, Shandong, China (approval no. 2019–01004), and all patients in the study sought the consent of their guardians and signed the “Informed Consent.”

### 2.2. Treatment Methods

The control group was treated with prednisone (2 mg/kg/d) orally for 8 weeks. 2–4 weeks after complete remission, they were treated with prednisone every other day after breakfast for 4 weeks, and the maximum amount should not be greater than 60 mg/d. After that, the maximum amount was reduced 2.5–5 mg every 2 to 4 weeks for 9 months of treatment. If recurrence occurs, the immunosuppressant cyclophosphamide should be added on this basis. The general dose is 2.0–2.5 mg/kg/d in 3 divided doses. The course of treatment is 8–12 weeks. The total amount should not exceed 200 mg/kg. (2) The therapy group was treated with Liuwei Dihuang Wan on the basis of the control group. Weight ≤25 kg: *Astragalus* 10 g, *Atractylodes macrocephala* 10 g, Chinese yam 12 g, Habitat 8 g, Paeonolium bark 8 g, Poria 10 g, Dogwood 10 g, *Alisma oriental*is 8 g, Dog ridge 10 g, *Cuscuta* 10 g, *Salvia miltiorrhiza* 8 g. Weight >25 kg: *Astragalus* 15 g, *Atractylodes macrocephala* 12 g, Chinese yam 15 g, Habitat 10 g, Dan bark 8 g, Poria 12 g, Dogwood 10 g, *Alisma orientalis* 8 g, Dog ridge 10 g, *Cuscuta* 15 g, *Salvia* 10 g. Children older than 12 years, 80–100/time, 2 times a day; children 7–12 years old, 50–80 ml/time, 2 times a day; and children 2–7 years old, 30–50 ml/time, daily 2 times.

### 2.3. Clinical Observation

#### 2.3.1. Safety Observation Indicators

  The general condition of the patients: heart rate, blood pressure, pulse, etc
  The blood routine, electrocardiogram, liver enzyme alanine aminotransferase (ALT), and aspartate aminotransferase (AST) tests were checked before and after the test

#### 2.3.2. Curative Effects Observation Index

1. 24-hour urine protein quantification: the 24-hour urine output was collected and the urine albumin was determined, which was tested by the laboratory of our hospital every two weeks.
2. ALB, triglycerides (TG), total serum cholesterol (TC), coagulation function-related indicators, creatinine, and urea nitrogen were tested by the laboratory of our hospital every four weeks.
3. Urine routine was tested once a week by the laboratory of our hospital.

#### 2.3.3. Efficacy Evaluation Criteria

1. Short-term curative effect: the urine protein of the children was observed after 8 weeks of treatment. (i) Complete remission: blood biochemistry and urine examination were completely normal, urine protein turned negative, and edema disappeared. (ii) Partial remission: urine protein was ++ or less, and edema disappeared. (iii) No remission: urine protein was +++ or more, and the edema remained unchanged from before treatment.
2. Long-term curative effect: (i) cure: the child was completely relieved, and the treatment was stopped for not less than 3 years. (ii) Improvement: except for the occasional small amount of proteinuria, other indicators were normal for more than 3 years. (iii) Invalid: urine protein was still +++ (or higher) or death [15].

#### 2.3.4. Safety Evaluation Standards

1. Safety assessment: Level 1: safe, without any adverse reactions; Level 2: relatively safe, if there were adverse reactions, do not need to do any treatment to continue the administration; Level 3: there are safety issues and moderate adverse reactions, and the drug could be continued after treatment; and Level 4: the test was discontinued due to adverse reactions.
2. Evaluation of the degree of adverse reactions: Mild: the symptoms of adverse reactions were mild, usually without treatment; Moderate: the symptoms of adverse reactions were obvious, important organs and tissues were damaged to a certain extent, and it was easy to recover; Severe: vital organs are damaged, and life is endangered.

#### 2.3.5. Observation Time and Follow-Up

(1) Continuous treatment for 12 weeks, and records of TCM symptoms and laboratory-related examination results were used as the basis for the short-term efficacy evaluation of the examination. After 12 weeks of treatment, those who still do not work should use other treatments or avoid delaying the disease. (2) Investigation and follow-up for half a year after the end of treatment, to record the recurrence of children with NS. (3) Recurrence rate was calculated using the following equation:

(1) $$\begin{array}{c} \text{recurrence rate}=\frac{\text{number of relapsed cases}}{\text{number of remission cases}}\times 100\%. \end{array}$$

### 2.4. Statistical Methods

Independent repetitions of experiments were 3 times. The detection index data were expressed as *x* ± *s* and analyzed by SPSS22.0 software (IBM, NY, USA). Differences between different groups were compared by analysis of variance or chi-square test, and the data between the two groups was tested by *t*-test. *p* < 0.05 was considered as statistically significant.

## 3. Results

### 3.1. General Conditions before Treatment

**Figure 1:**
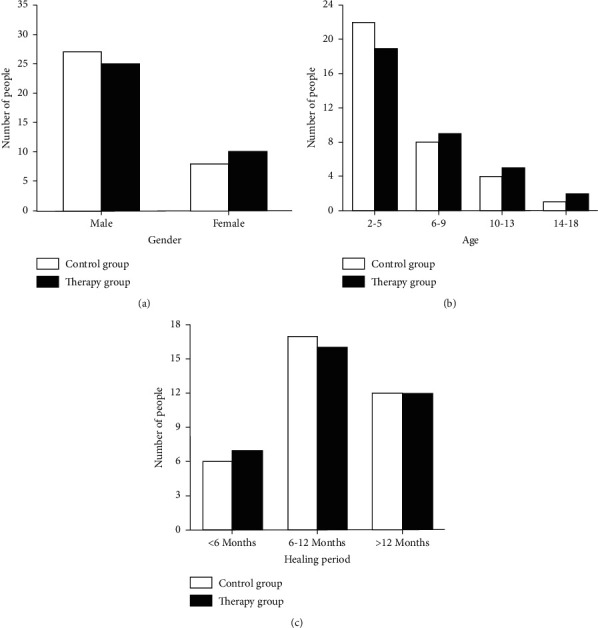
General conditions before treatment. (a) The gender distribution in the control group and therapy group. (b) The age distribution in the control group and therapy group. (c) The length of disease distribution in the control group and therapy group.

The basic conditions between the two groups were compared and results showed that the two groups had no statistical difference in gender distribution, age distribution, and length of disease distribution before treatment, and they were comparable (Figures 1(a)–1(c)).

### 3.2. Comparison of the Levels of 24 h UPro in the Two Groups (g/24 h)

As shown in Table 1, there was no significant difference between the therapy group and the control group in the 24 h UPro before treatment (*p* > 0.05). However, the therapy group and the control group have higher UPro after treatment compared with before treatment (^#*∗*^*p* < 0.05). Moreover, the therapy group had a better curative effect in reducing 24 h UPro than the control group after treatment (^*∗*^*p* < 0.05).

### 3.3. Comparison of the ALB Level between the Two Groups (g/L)

Results from Table 2 revealed that the ALB level in the therapy group and the control group was 20.54 ± 1.72 and 20.43 ± 1.41, and there was no significant difference between the therapy group and the control group before treatment (*p* > 0.05). After treatment, the ALB level in the therapy group (37.92 ± 3.62) and the control group (31.08 ± 1.95) increased significantly (^#*∗*^*p* < 0.05), indicating that the treatment was effective. The level of ALB in the therapy group was higher than that of the control group, suggesting that the combination of western medicine and TCM in the therapy group has a better effect on the improvement of hypoalbuminemia (^*∗*^*p* < 0.05).

### 3.4. Comparison of the Levels of Triglyceride and Cholesterol between the Two Groups

The triglyceride level of the therapy group and the control group before treatment was 7.31 ± 1.95 and 7.45 ± 2.07, and the cholesterol level was 3.21 ± 0.57 and 3.17 ± 0.63, respectively. There was no significant difference between the control group and the therapy group in the two indexes of blood lipid (*p* > 0.05), and they were comparable. The levels of triglycerides and cholesterol in the therapy group and the control group after treatment were lower than those before treatment (^#^*p* < 0.05), indicating that the treatments of both groups were effective, and the triglyceride and cholesterol levels in the therapy group decreased more as compared to the control group (^*∗*^*p* < 0.05), indicating that the therapy group was better than the control group in improving the patient's blood lipid status (Table 3).

### 3.5. Comparison of the Coagulation Function between the Two Groups

There was no difference in coagulation between the therapy group and the control group before treatment (*p* > 0.05), which was comparable. After treatment, the fibrinogen and D-dimer in both groups were significantly improved (^#^*p* < 0.05), and the effect of the combined Chinese and Western medicine therapy group was significantly better than that of the control group. However, there was no significant difference between the two groups in prothrombin time and activated partial thrombin time (Table 4).

### 3.6. Comparison of the Renal Function between the Two Groups

The children's renal function was evaluated by the creatinine and urea nitrogen levels. Results showed that the creatinine and urea nitrogen levels of the control group and therapy group were not significantly different (*p* > 0.05), which was comparable. After treatment, the creatinine and urea nitrogen levels of the patients in the therapy group and the control group were decreased, but not significantly (^#^*p* > 0.05), indicating that the renal function of the two groups had no notable changes after treatment. There was no significant difference in renal function between the two groups after treatment (^*∗*^*p* > 0.05), which further indicated that there was no renal toxicity after TCM combined with hormone therapy, and this treatment method was safe and could be used in clinical treatment (Table 5).

### 3.7. Comparison of the Related Immune Indexes between the Two Groups

**Figure 2:**
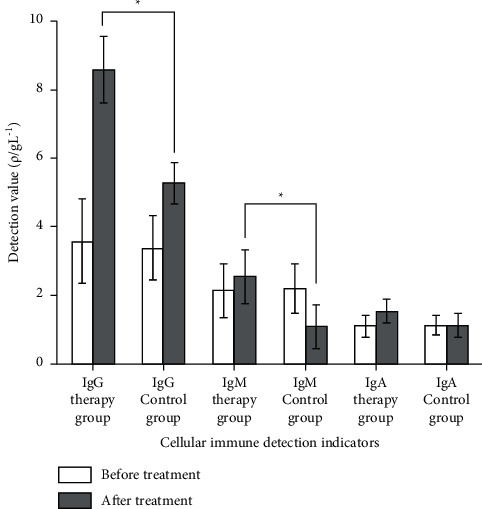
Comparison of cellular immunoassay between the two groups before and after treatment.

**Figure 3:**
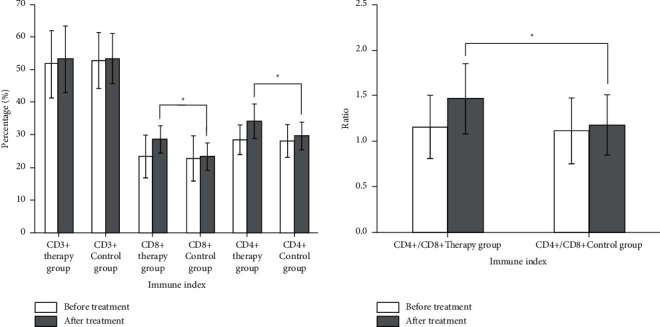
Comparison of humoral immune indexes before and after treatment between the two groups.

There was no significant difference in T lymphocyte population and humoral immunity levels between the two groups of children before treatment. The cellular immunity and humoral immunity levels of the therapy group were significantly higher than those of the control group (*p* < 0.05) (Figures 2 and 3). Moreover, the infection rate of the therapy group was significantly reduced. At the same time, the indicators of immunoglobulin and T cell subgroups increased significantly after the application of TCM, suggesting that PNS immunodeficiency has been effectively improved, thereby reducing the side effects of hormones and immunosuppressants.

### 3.8. Comparison of Curative Effect between the Two Groups

The children were followed up for six months. Among them, 3 cases were lost to follow-up in the control group, 2 cases were missed to follow-up in the therapy group, and the missed cases were dropped from investigation. The curative effects of the two groups are shown in Tables 6 and 7, where the total effective rate of the therapy group was higher than that of the control group (*p* < 0.05), suggesting that in the therapy group better outcomes were achieved.

### 3.9. Security Comparison

**Figure 4:**
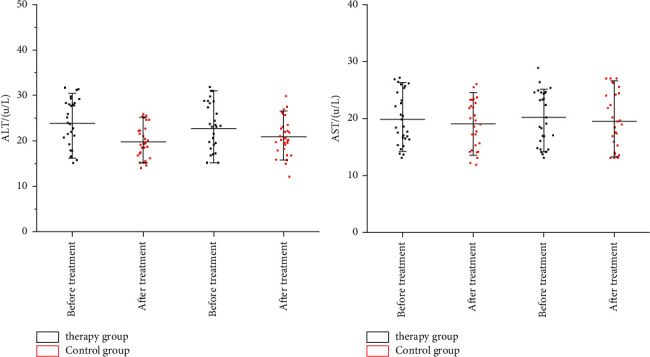
Comparison of changes in liver function between the two groups (*μ*/L).

As shown in Figure 4, there is no significant difference in liver function between the therapy group and the control group before treatment (*p* > 0.05), which is comparable. The two important indexes of liver function in the therapy group and the control group have no significant changes (*p* > 0.05). Moreover, there was no statistically significant difference between the two groups before and after treatment (*p* > 0.05).

**Figure 5:**
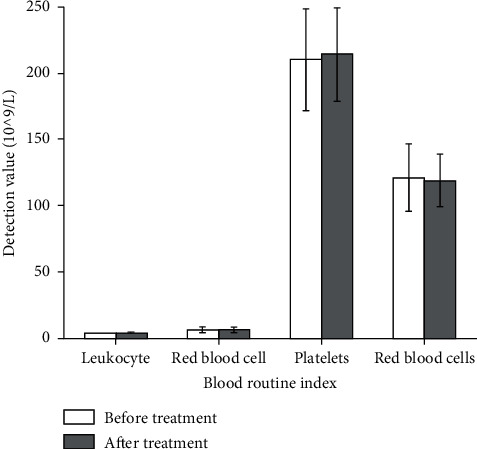
Comparison of blood routine between the two groups.

Furthermore, results from Figure 5 showed that there is no difference between the therapy group and the control group in blood routine before treatment (*p* > 0.05), which is comparable. There was no significant difference in the two important liver function indexes before and after treatment in the therapy group and the control group (*p* > 0.05). Moreover, there was no statistical difference between the two groups after treatment (*p* > 0.05), indicating that the therapy group and the control group had no difference in blood routine effects before and after treatment. These results suggested that neither the therapy group nor the control group had any obvious adverse reactions during the experiment. Although in some children mild digestive system reactions were observed after taking this drug, they could be relieved by themselves or corresponding interventions, without affecting the trial process.

### 3.10. Comparison of Recurrence Rate between the Two Groups

After the patients were treated, the children with effective cases in the two groups were followed up for 6 months. Results showed that there were 33 effective cases in the Chinese medicine decoction combined with the hormone therapy group, 7 relapses, and the recurrence rate in the therapy group is 21.1%. In the hormone therapy group alone, there were 32 effective cases, 17 relapsed, and the recurrence rate was 53.1%, suggesting that the recurrence rate of the Chinese medicine decoction combined with the hormone therapy group was significantly lower than that of the control group, and the difference was statistically significant (*p* < 0.05) (Table 8). These results indicate that the Chinese medicine decoction combined with hormone treatment has a good effect in PNS.

## 4. Discussion

Previous studies revealed that many hormonal Western medicines used to treat primary nephrotic syndrome could cause adverse reactions such as osteoporosis, digestive system infections, lipid metabolism disorders, atrophy of the adrenal cortex, and hypofunction [16]. However, the side effects of the drug can be reduced by adding TCM that strengthens the spleen and qi and nourishes the Yin and kidney. The main mechanism of TCM is to allow corticosteroids (GC) to combine with GCR to exert pharmacological effects. Clinical trials have shown that TCMs such as *Astragalus* can increase the number of GCR and increase the effective combination of GC and GCR. This kind of TCM can alleviate the disease of Yang hyperactivity and Yin deficiency, reduce the adverse reaction of hormone drugs on the immune mechanism in children, improve the immune function of the body, and play a good role in reducing recurrence, consolidating curative effect, preventing cold, and playing a positive role in the treatment of diseases.

*Astragalus* is a tonic TCM with the functions of invigorating qi and promoting yang, promoting hydration, reducing swelling, and strengthening the surface and antiperspirant. Its active ingredients are mainly *Astragalus* polysaccharides, flavonoids [17], and saponins [18], which have various pharmacological effects such as regulating immune function, antitumor, regulating body metabolism, and antiaging. The pharmacological effects of astragalus aqueous extract in adriamycin nephropathy improved the state of high coagulation and high viscosity of blood and the coagulation time, (1) avoiding the formation of thrombi; (2) increasing the number of CD3+ lymphocytes in vivo, increasing the ratio of CD4+/CD8+, and improving the immune function; (3) improving urinary albumin and increasing plasma albumin level; (4) reducing total cholesterol and triglyceride levels, improving endogenous creatinine clearance rate, urea nitrogen, and other renal function indexes; (5) relieving edema. *Astragalus* plays a role in the intervention of nephropathy, such as supplementing qi and strengthening surface, supplementing qi of spleen and lung, supplementing kidney and supplementing qi, and benefiting water in reducing swelling. In addition, in clinical use of *Astragalus* and Western medicine intervention, nephrotic syndrome can reduce the adverse effects of chemical drug intervention.

In this study, glucocorticoids combined with TCM were used for the comprehensive treatment of primary nephrotic syndrome in children, and results showed that the urinary protein quantification, triglycerides, cholesterol levels, and children's recurrence rate were significantly lower than those of the control group, and the levels of total protein and albumin, immune indexes, and blood coagulation function were significantly higher than those of the control group. The comparison of the renal function indexes, liver function indexes, and blood routine concluded that there is no noticeable difference in the incidence of adverse reactions between the two groups.

## 5. Conclusion

In summary, the use of hormones and TCMs to treat children with primary nephrotic syndrome can effectively reduce the adverse reactions caused by hormone therapy, improve therapeutic outcomes, and reduce the recurrence rate. Moreover, it could improve the sensitivity of children to hormones and further shortens the hormone-induced remission time, and it has a better synergistic effect in the use of hormones in the treatment of primary nephrotic syndrome in pediatric patients.

## Figures and Tables

**Table 1:** Comparison of 24 h UPro level between the two groups (g/24 h).

Group	Before treatment	After treatment
Therapy group	4.51 ± 1.73	0.76 ± 0.42^#*∗*^
Control group	4.63 ± 1.77	1.74 ± 0.69_#_

^#^ *p* < 0.05 vs. after treatment; ^*∗*^*p* < 0.05 vs. therapy group.

**Table 2:** Comparison of the ALB level between the two groups (g/L).

Group	Before treatment	After treatment
Therapy group	20.54 ± 1.72	37.92 ± 3.62^#*∗*^
Control group	20.43 ± 1.41	31.08 ± 1.95_#_

^#^ *p* < 0.05 vs. after treatment; ^*∗*^*p* < 0.05 vs. therapy group.

**Table 3:** Comparison of the levels of triglyceride and cholesterol between the two groups (mmol/L).

Blood lipids	Group	Before treatment	After treatment
Triglycerides	Therapy group	7.31 ± 1.95	4.85 ± 1.37^#*∗*^
	Control group	7.45 ± 2.07	5.73 ± 1.84^#^
Cholesterol	Therapy group	3.21 ± 0.57	1.82 ± 0.53^#*∗*^
	Control group	3.17 ± 0.63	2.31 ± 0.71^#^

^#^ *p* < 0.05 vs. after treatment; ^*∗*^*p* < 0.05 vs. therapy group.

**Table 4:** Comparison of the coagulation function between the two groups.

Coagulation function	Group	Before treatment	After treatment
Prothrombin time (s)	Therapy group	11.4 ± 1.6	12.7 ± 2.0
	Control group	11.9 ± 1.8	12.0 ± 1.5
Activated partial prothrombin time (s)	Therapy group	31.0 ± 2.7	33.6 ± 2.4
	Control group	31.8 ± 3.8	36.1 ± 4.3#
Fibrinogen (g/L)	Therapy group	5.09 ± 1.1	2.13 ± 0.8^#*∗*^
	Control group	5.15 ± 1.3	2.72 ± 0.8^#^
D-dimer (ug/ml)	Therapy group	1.42 ± 0.4	0.44 ± 0.2^#*∗*^
	Control group	1.37 ± 0.4	0.71 ± 0.3^#^

^#^ *p* < 0.05 vs. after treatment; ^*∗*^*p* < 0.05 vs. therapy group.

**Table 5:** Comparison of the renal function between the two groups.

Kidney function	Group	Before treatment	After treatment
Creatinine (*μ*mol/L)	Therapy group	104.33 ± 24.34	95.92 ± 20.53^#*∗*^
	Control group	106.79 ± 29.78	102.03 ± 22.93^#^
Urea nitrogen (mmol/L)	Therapy group	7.23 ± 2.97	6.46 ± 3.14^#*∗*^
	Control group	7.17 ± 3.15	6.45 ± 3.36

^#^ *p* < 0.05 vs. after treatment; ^*∗*^*p* < 0.05 vs. therapy group.

**Table 6:** Comparison of recent treatment courses between the two groups (*n* (%)).

Group	*n*	Complete response	Some relief	No relief	Total effective rate (%)
Therapy group	32	11 (34.4%)	13 (40.6%)	8 (25.0%)	75.0
Control group	33	17 (51.5%)	14 (42.4%)	2 (6.1%)	93.9

**Table 7:** Comparison of recent treatment courses between the two groups (*n* (%)).

Group	*n*	Cure	Get better	Invalid	Death	Total effective rate (%)
Therapy group	32	7 (21.9%)	16 (50.0%)	8 (25.0%)	1 (3.1%)	71.9
Control group	33	18 (54.5%)	14 (42.4%)	1 (3.0%)	0 (0.0%)	97.0

**Table 8:** Comparison of the recurrence rate between the two groups.

Group	Case	0	1	2	≥3 times	Recurrence rate (%)
Therapy group	33	26	4	3	0	21.1
Control group	32	15	7	6	4	53.1

## Data Availability

The datasets used and/or analyzed during the present study are available from the corresponding author upon reasonable request.
